# What Is New in Glaucoma: From Treatment to Biological Perspectives

**DOI:** 10.1155/2021/5013529

**Published:** 2021-04-14

**Authors:** Raffaele Nuzzi, Paola Marolo, Alessia Nuzzi

**Affiliations:** ^1^Eye Clinic Section, Department of Surgical Sciences, University of Turin, Turin, Italy; ^2^Department of Clinical Sciences and Community Health, Eye Clinic San Giuseppe Hospital,IRCCS Multimedica, University of Milan, Milan, Italy

## Abstract

Glaucoma is a chronic silent disease and an irreversible cause of blindness worldwide. Research has made many efforts to improve disease control and especially to anticipate both early diagnosis and treatment of advanced stages of glaucoma. In terms of prevention, networking between professionals and nonprofessionals is an important goal to disseminate information and help diagnose the disease early. On the other hand, the most recent approaches to treat glaucoma outcomes in its advanced stages include electrical stimulation, stem cells, exosomes, extracellular vesicles, and growth factors. Finally, neuronal plasticity-based rehabilitation methods are being studied to reeducate patients in order to stimulate their residual visual capacity. This review provides an overview of new approaches to future possible glaucoma treatment modalities and gives insight into the perspectives available nowadays in this field.

## 1. Introduction

Glaucoma is one of the leading causes of blindness in the world, second only to cataracts [1, 2]. It is a chronic, degenerative disease affecting the optic nerve, but insidious: in fact, when the etiopathogenetic process has started and has already damaged the nerve fibers, the symptomatology is almost silent [3, 4]. When the patient becomes aware of the visual impairment, the neural function is already compromised and the chances of recovery are significantly reduced. There is no scientific evidence of the field of visual recovery once its defect has been documented. However, a perimetric learning effect among a percentage of patients could occur, very unlikely to represent a real improvement. For these reasons, glaucoma is a disease of enormous social impact, both from the human point of view, because it is highly disabling and compromises the quality of life and autonomy of those affected [5], and from an economic point of view: for its clinical-therapeutic management, in fact, a substantial percentage of public healthcare expenditure is invested [6]. The term “glaucoma” actually encompasses several forms of optical neuropathies with still partly obscure etiopathogenesis associated with typical visual field alterations and increased intraocular pressure [7, 8]. In reality, this last characteristic is not the rule: in recent years, the number of cases of “normotensive glaucoma,” which is not associated with an increment in IOP, has increased dramatically, especially in relation to the lengthening of life expectancy [9, 10]. In fact, it has long been known that only about half of the glaucoma cases have intraocular pressure above reference values [11–13]. In any case, the most accredited etiopathogenetic hypothesis would be the death of retinal ganglion cells due to mechanical stress and apoptosis following ischemic and/or chemical mechanisms, which would seem to have glutamate and NMDA receptor activation as protagonists, which would cause an exponential increase in intracellular calcium concentration, thus triggering irreversible damage to DNA and cell death [14, 15]. In light of this, it is clear that it is of great importance to study the phases of this disorder and to continually seek new preventive and therapeutic strategies [16]. According to the current scientific panorama, the therapeutic possibilities are aimed at acting both on the initial stages of the disease and on the final outcomes, i.e., on the advanced stages of glaucoma for which the lesions are considerable and no longer reversible, but in which it is possible to intervene by enhancing the residual functions at the highest level. In this context, the aim of our manuscript is to provide a comprehensive review of the recently investigated new approaches to treat early and late stages of the disease.

This literature search was performed in accordance with the Preferred Reporting Items for Systematic Reviews and Meta-Analyses (PRISMA) guidelines [17]. Studies reporting novel treatment strategies of glaucoma were systematically reviewed. PubMed, MEDLINE, Web of Science, and Embase databases (from inception up to 1 January, 2020) were searched. The electronic search method included the terms ‘glaucoma', ‘novel therapies', ‘electrical stimulation', ‘micropulse', ‘stem cells', ‘exosomes', ‘optic nerve regeneration', ‘growth factors', ‘rehabilitation', which were connected in various combinations by ‘or'/'and'. The last search was carried out on 1 January, 2020. Either prospective or retrospective, both randomized and not randomized studies were considered for eligibility. No restrictions in terms of follow-up were applied. Eligible papers must have been published in peer-reviewed journals and in English, with no publication date or publication status limitations. Titles and abstracts of all identified studies were independently reviewed by two researchers (P.M. and A.N.) to assess eligibility. A full-text evaluation of all potential studies was performed later. Once studies have been selected and included, data were extracted by two independent investigators (P.M. and A.N.). When discrepancies were found, a third author (R.N.) was involved to achieve consensus.

## 2. Overview of the Current Evolution of Treatment Strategies

In addition to the well-known hypotensive drugs, in recent years, research has focused on the development of new local and systemic drugs to be used to reduce intraocular pressure. The main innovative drugs discovered are latrunculin derivatives, ROCK inhibitors, cannabinoids, local calcium channel blockers, and A1 receptor agents. Latrunculin derivatives have mild hypotonic properties and low solubility [18], while ROCK inhibitors have been shown to be effective in reducing pressure in animal models, also acting as neuroprotectors and vasoregulators [19, 20]. These agents seem to interfere with the healing that generally occurs after filtering surgery [21]. Cannabinoids increase the outflow of aqueous humor that acts on ciliary processes with a vasodilator effect and increase PGE2 levels [22–24]. A1 receptor agonists [25] and selective calcium channel blockers improve watery mood drainage, but the latter can lead to serious systemic consequences, such as severe bradycardia and arterial hypotension [26].

Moreover, in the last years, great interest has been devoted to the design of modern devices for instilling drugs in situ: one of the main disadvantages of eye drops treatment is, in fact, the poor compliance and fluctuating adherence of patients to the therapy [27]. These new delivery systems include eye inserts, surgical implants, soft medicated contact lens, and nanospheres. Ocular inserts are projected to provide medication for several days, while surgical implants can perform their function for months but require surgical intervention [28]. Another product in the design phase is the medicated soft contact lens, which, however, must be worn constantly and does not always allow drugs to pass through the surface of the eye in adequate quantities [27, 29]. Nanospheres are another type of device that ensures good drug penetration but does not prevent the patient's from poor adherence to therapy because these microspheres are administered through eye drops [30]. To date, the experimental data in favor of the application of these delivery systems are promising but still limited, and further studies are needed to confirm such evidence [31].

Concerning laser therapy, diode laser trabeculoplasty (DLT) uses lower energy spots than SLT (selective laser trabeculoplasty) and ALT (argon laser trabeculoplasty) for the same performance, and micropulse diode laser trabeculoplasty (MDLT) emits microspots to limit heat-induced damage to adjacent structures [32]. Ab intero excimer laser trabeculectomy is based on the creation of microperforation connecting the anterior chamber to the Schlemm's channel, theoretically not causing heat-induced damage and healing [33].

However, its effectiveness is still uncertain. Despite the advantages of laser technology, research is still focusing on finding the best way to minimize tissue rupture and subsequent healing and to achieve better effectiveness in terms of lowering IOP.

Regarding surgery, the main goal of the latest research has been to improve its risk/benefit ratio, trying to overcome the traditional trabeculectomy technique, which still remains the gold standard of treatment. Minimally invasive glaucoma surgery (MIGS) [34] has been developed in an attempt to obtain a better efficacy/safety ratio in eyes with mild or medium-mild grade glaucoma. The efficacy of MIGS is lower in IOP reduction compared to standard surgery, and its costs are elevated; nevertheless, it is a safe technique and can play a role in a subgroup of patients who are not willing to undergo regular surgery or when patients with a moderate level of pressure lowering do not tolerate drops or do not respond to laser treatment.

## 3. Novel Treatment Strategies for Glaucoma Outcomes

Despite the numerous therapeutic efforts described above, glaucoma is often diagnosed late and blocking the natural evolution of the disease is still the main obstacle in its management. Delaying therapies to the more advanced stages of glaucoma leads to its evolution towards irreversible optic nerve damage and blindness. In this respect, numerous studies have examined the action of new molecules and techniques to improve the control of the disease and restore lost nerve function and protect its anatomical and functional residuals. It is possible to intervene both separately and concurrently on four levels: the trabecular meshwork, the ciliary body, the retinal ganglion cells (RGCs), and the optic nerve. The main approaches concern the use of stem cells, exosomes or extracellular vesicles, neuroprotection, and rehabilitation therapy. As far as the optic nerve is concerned, the most encouraging instruments are growth factors and chitosan sheaths.

In this section, we will focus our attention on the latest discoveries on the treatment of glaucoma, explaining for each strategy which aspects of the disease it targeted, its underlying mechanisms and/or molecules, its development phase, and the main obstacles to be overcome in order to bring it to the clinic.

### 3.1. Electrical Stimulation of the Trabecular Meshwork

Electrical stimulation has recently been proposed as a novel approach to decrease IOP in open-angle glaucoma [35]. The target of this technique is the trabecular meshwork (TM), which is not just a passive way of drainage of the aqueous humor but also has an active role in the resistance to the passage of AH through mechanisms that are not fully understood [36]. Early transcorneal electrical stimulation (TcES) [37] has been shown to have a positive IOP lowering effect in preclinical studies. RGCs in the eyes of gerbil prone to retinal lesions related to acute ocular hypertension have been protected from damage by TcES. The implicit mechanism of action was the modulation of the inflammatory response activated by microglial cells [37]. Transpalpebral electrical stimulation (TES) performed on human eyes with open-angle glaucoma has been shown to have a significant effect in lowering IOP [35]. The purpose of TES is to reproduce the role of tyrosine kinase inhibitors by stimulating the reactivation of calcium-activated potassium channels in TM cells. The hyperpolarization induced by the efflux of potassium to TM promotes its relaxation and thus facilitates the outflow of aqueous humor to Schlemm's channel. The progressive functional damage of the TM in glaucoma is inversely proportional to the effectiveness of electrical stimulation. When the ion channel dysfunction is too advanced and both the volume and the elasticity of TM cells are affected, it is more difficult to obtain a good response. Less trabecular function in more advanced glaucoma results in reduced efficacy of the procedure and increased need to replicate it. Therefore, it is our opinion that electrical stimulation may be more useful in the early stages of the disease. Additional studies are needed to further investigate this new technique and to evaluate the maintenance of the IOP lowering effect in time after treatment.

### 3.2. Micropulse Cyclophotocoagulation and Ultrasound Cyclomodification

Cilioablation is a well-known procedure that has undergone a drastic evolution in basic technology in recent years. While prostaglandin analogues activate the receptors of the smooth muscles of the ciliary body and increase the uveoscleral outflow, surgical ablation of a ciliary body part can decrease the secretory activity of the ciliary epithelium, thus reducing IOP. Diode laser cyclophotocoagulation (CPC) has shown an encouraging risk-benefit profile, with a much more tolerable side effect profile than previous cyclocryotherapy and has led to the development of transscleral diode CPC and endoscopic diode CPC.

Advances in the study of diode technology have allowed the development of the new transscleral Micropulse Diode laser CPC. Its diode laser emits a series of short (microsecond), repetitive bursts of energy, so that the thermal effect is limited to the absorbing tissue with minimal heat diffusion to adjacent structures. During the cooling period, the tissue has time to relax and return to the base temperature. Micropulse diode laser technology has been successfully used for the treatment of diabetic retinopathy and maculopathy, and the expectation on glaucoma is to achieve the same IOP lowering effect as traditional CPC diode, with fewer associated side effects. Tan et al. [38] conducted a study in which good rates of IOP and med reduction were detected and about one-third of the patients reported suffering pain during the procedure, while none described the discomfort as moderate or severe. Although the initial results are encouraging, further studies with longer follow-up are required to better assess the actual benefits of CPC micropulse compared to traditional CPC. Efforts in the search for an alternative to cyclodestructive procedures to further reduce tissue damage have led to the introduction of high-intensity ultrasound (HIFU) for the treatment of glaucoma. The device, firstly proposed in 1991 [39], has recently been redesigned into a compact and easy-to-use device (EyeOP1, EyeTechCare, Rillieux-la-Pape, France). The two essential components of the system are the generator, which gives power to piezoelectric transducers, and the pressure reduction system, which modulates the suction of the probe with its ultrasonic beam. The device uses what is known as circular ultrasound cyclocoagulation and simultaneously treats the entire ciliary body through the release of a titratable dose of six distinct ultrasonic energy beams. In the first clinical study on this procedure [40], a good response to IOP lowering was obtained in the treated group with a duration of four seconds of ultrasound exposure per shot and the complications were three cases of superficial punctate epitheliopathy and one of central ulcer, with no reports of chronic pain hypotony or phthisis bulbs.

Considering that cyclodestructive procedures have been used to treat the later stages of glaucoma, particularly neovascular glaucoma, it is not surprising that the results are mostly poor in the literature. Recently, some studies have evaluated the use of the new technologies described to treat earlier stages of glaucoma, and this has been possible thanks to their good safety profile [41, 42]. With the increase in supporting evidence, more and more surgeons are now considering these new technologies in the treatment of early stages of glaucoma rather than more advanced cases.

### 3.3. Stem Cells Therapy

Stem cells are an important resource for the maintenance, repair, and possible regeneration of anatomical structures such as the optic nerve [43]. The scientific interest in these cells is due to their unique properties, including the capacity to divide themselves an infinite number of times and the ability to differentiate into many types of cells. However, ethical concerns and technical barriers are still implied. Promising results have been reported in the literature, but further research is required in order to bring its application to the clinic. To date, the most used cell lines are adult limbic stem cells to restore the corneal epithelium [44], those situated at the location of Schwalbe's ring (the transitional zone between the corneal endothelium and the TM) [45, 46] and those of the ciliary epithelium, which seem to be able to differentiate into various retinal cell strains [47–49]. With regard to differentiation into neural and retinal cells, embryonic/progenitors retinal stem cells have demonstrated successful differentiation into retinal cell types, either in vitro [50] or in vivo [51]. Nevertheless, their use for ex vivo cell therapy still presents barriers: insufficient availability of stem cells/progenitors, immune rejection, and clinical issues related to embryonic and fetal origins. To overcome this hurdle, Parameswaran et al. [48] have demonstrated that mouse fibroblast induced pluripotent stem cells (iPSCs) were also able to generate RGCs, rods, cones, and photoreceptors. The iPSCs were stimulated by a simulated microenvironment of late retinal histogenesis and finally expressed retinal cell type-specific regulators. Anyway, the clinical application of iPSCs for cell therapy in glaucoma is still unknown, and in order to consider the use of stem cells as a replacement for RGCs, some challenges should be addressed. First, the stimulation method of transplanted stem cells is not yet fully understood and further studies are needed in order to bring functional results to the damaged optic nerve in glaucomatous eyes. In addition, RGCs have a heterogeneous nature with different morphological and molecular criteria, making the induced differentiation progress even more challenging. Other types of stem cells that can be employed are mesenchymal stem cells (MSCs) from bone marrow and adipose tissue [52]. The main advantages of MSCs are their pluripotency, their ease of extraction, and their availability for autologous transplantation. The neuroprotective effects of MCSs in experimental glaucoma are now gaining more and more evidence in experimental glaucoma models [52, 53]. MSCs have also shown the ability to produce neurotrophic factors, such as brain-derived neurotrophic factor (BDNF) and ciliary neurotrophic factor (CNTF), with a 28% reduction of cell death after 1 month from glaucoma onset [54]. Connick et al. [55] have suggested a promising application of MSCs that demonstrates positive effects in patients with multiple sclerosis after their intravenous administration. Visual acuity and visual evoked potential latency have improved. It would be interesting to evaluate the possibility of reintegrating trabecular cells by transplanting MSCs into the anterior chamber. The hydrodynamics of aqueous humor should allow the cells to settle at the iridocorneal level, with phenomena of differentiation and intratissue migration. However, experimental data supporting their application in glaucoma are few, except for the production of neurotrophic factors by transplanted stem cells that stimulate ganglion cells survival.

### 3.4. Exosomes or Extracellular Vesicles

Recent studies have demonstrated the paracrine capacity of MSCs to secrete exosomes [56–58]. Exosomes small extracellular vesicles of endocytic origin of 30–100 nm diameter, with a membrane enclosure and containing proteins, as well as mRNA and miRNA, which can be delivered to the nearby cells. The advantages of exosomes are many. First, they can be easily isolated and purified; second, they are cell-free and do not proliferate, thus avoiding ethical issues related to stem cells. They can also be easily stored and the application of specific doses is easier. Since they are really small, they can migrate into the ganglion cell layer from the vitreous, and this is not possible with transplanted cells. Furthermore, and most importantly, exosomes are immunologically inert.

It has been proven that the content of exosomes is translated to other cells when the exosome blends with the nearby cell membranes and this leads to the translation of new proteins [59]. Once the exosomes deliver their content to cells, this is shuttled inside endocytic vesicles and delivered to endoplasmic reticule and lysosomes [60]. Mead and Tomarev isolated the exosomes from stem cells derived from bone marrow MSC (BMSC) and tested them into a murine model [61]. They performed the first study in which RGC were treated with exosomes and, for the first time, BMSC-derived exosomes were delivered into an eye. In their study, a significant neuroprotective effect was shown when the rapt optic nerve model was analyzed with optical coherence tomography, electroretinography, and immunohistochemistry. RGC survival, regeneration of axons, and partial prevention of RGC axon loss (measured as RNFL thickness) and preservation of RGC function (measured with electroretinogram) were possible thanks to intravitreal injections of BMSC-derived exosomes and were associated with miRNA-dependent mechanisms.

However, the miRNA content and its targets have to be better characterized, and it is not clear which dose of intravitreal exosomes should be injected in order to have a therapeutic effect (weekly, biweekly, or monthly). Further research is needed to bring these promising new results into the clinic.

### 3.5. Optic Nerve Axonal Regeneration

Irreversible lesions related to the progression of glaucoma lead to optic nerve atrophy and loss of visual functions up to blindness. It is not necessary to emphasize how important it is to avoid such an eventuality and how much effort and energy research have invested for years to discover therapeutic strategies to be adopted in this context.

One of the most investigated therapeutic approaches is optic nerve transplantation, which can be achieved with a peripheral nerve graft, but currently, the most promising resource seems to be the realization of polymeric membranes. The optic nerve transplantation is not yet ready to be applied in human models, and further studies are needed; anyway, many researchers are now focusing on the development of this topic.

After any kind of optic nerve injury, the regeneration of the optic nerve is blocked by major obstacles. The most considerable ones are the following: apoptosis of RGC, the difficulty in triggering the axonal growth, and the presence of inhibitory factors in the microenvironment of the central nervous system. Concerning optic nerve regeneration, a promising strategy is the creation of conduits that damaged neurons and axons, which can be utilized as a guide for nerve repair and regeneration; such conduits can be of various kinds, and many studies are now focusing on chitosan, a promising derivative of chitin extracted from shellfish [62–64]. Peripheral nerve grafts demonstrated an effect on the restoration of the pupillary reflex in mice with the damaged optic nerve. In addition, other substrates are under investigation. A peptide nanofiber scaffold has been studied in hamsters, with good recovery of visual function. Negishi et al. [65] applied a silicone tube graft enriched with purified Schwann cells, extracellular matrix, and growth factors in mice subjected to axotomy, showing regeneration of blood vessels, RGC, and axons. Concerning chitosan, studies demonstrated their utility in neural regeneration of either the peripheral or the central nervous system [63]. In order to facilitate neural regeneration, chitosan can be enriched with adhesion molecules, MSCs, and neurotrophic factors. Polyglycolic acid- (PGA-) chitosan scaffolds [66] and cationic chitosan-graft-poly(*ε*-caprolactone)/polycaprolactone (CS-PCL/PCL) scaffolds have been studied with promising results concerning their potential in stimulation and regeneration of damaged nerve fibers.

Triggering the neuronal growth implies the simultaneous action on different intracellular signals [67]. One of the most interesting pathways of triggering involves the ROCK inhibitors, which have a negative effect on the ROCK signaling cascade (which is itself a negative regulator of neural growth). In addition, alpha-crystallin proteins, which are components of the ocular lens, showed antiapoptotic properties thanks to their structural homology with heat shock proteins with chaperone-like features [68]. Piri et al. [69] demonstrated decreased levels of these proteins in glaucoma models, leading to the thought that downregulation of these proteins may reduce RGC survival. Other studies [68] supported this theory and further research is ongoing in order to give some clinical application to this interesting finding.

Molecular targets have been investigated in the field of new glaucoma treatment strategies [70]. Death from RGC is related to neurotrophic factor deprivation, hypoxia, excitotoxicity, gene dysregulation, and activation of apoptosis. Therefore, studies have focused on the possibility of enhancing the BDNF-TrkB signal (brain-derived neurotrophic factor-tyrosine protein kinase) and on the chance of pharmacologically modulating TrKB. Endogenous phosphatase Shp2 was also studied, considering its role in regulating TrKB. Recent findings have shown that stress-induced protein aggregation could cause the formation of unfolded proteins in the ER (endoplasmic reticulum) and apoptosis. In order to modulate the equilibrium between the apoptotic and the survival pathways, also proapoptotic Bcl2 and antiapoptotic Bax molecules are under investigation.

Furthermore, many studies focused on the beneficial effect of local, controlled inflammation. Vitreal inflammation induces the activation of retinal astrocytes and Müller cells and the secretion of many glial-derived growth factors, including BDNF, CNTF, leukemia inhibitory factor (LIF), and bone morphogenetic proteins (BMP) [71]. This event promotes neuroprotection of the RGCs and axonal growth by interacting with macrophage-derived factors (MDF), suggesting that immunomodulatory treatments may promote optic nerve regeneration. Intravitreal injections of zymosan and other immunoregulatory molecules, as well as the release of *β*/*γ*-crystallins from the injured lens [72], proved a proregenerative effect on the optic nerve. Zymosan is a yeast cell wall carbohydrate and it is a toll-like receptor 2 (TLR-2) ligand, which stimulates the ingress of the macrophages into the vitreous body and the MDF production when injected inside the vitreous [73]. Optic nerve regeneration was also shown using another TLR-2 ligand, the Pam_3_Cys, which is a water-soluble bisacyl-lipopeptide and a selective TLR-2 agonist. Its intravitreal application can induce glial activation, transform RGCs into a regenerative state, and stimulate axon regeneration [74].

### 3.6. Neurotrophic Growth Factors

As previously mentioned, particular attention has been dedicated to oxidative stress and its etiopathogenetic role in glaucoma. In fact, it has been observed that ocular hypertension establishes a stress condition that stimulates oxygen-free radicals production, which harms both directly and indirectly the retinal cells [75]. The use of molecular agents capable of arresting this oxidative burst would therefore be desirable. Currently, only brimonidine seems to possess neuroprotective properties and it has been shown that in patients treated with this substance, there is a slowdown in campimetric damage compared to those treated with timolol [76]. Another promising molecule is citicoline, already approved in Italy, i.e., a molecule previously involved in other neurodegenerative diseases, which could be used as a therapeutic tool in addition to hypotonic pharmacological treatment. Other interesting molecules are EPO (erythropoietin), BDNF, and CNTF, which appear to be involved in the growth and survival of RGCs: they are administered intravitreally, currently, their action is transient, and their use may cause teratogenic ocular effects. The most desirable resource remains gene therapy, which would directly induce endogenous production of these neurotrophic factors without the need to inject them externally [70]. Gene therapy can also take advantage of siRNA and polysaccharide or liposomal nanoparticles that act as vectors for placing a particular gene at a specific site [77]. The approach via viral vectors has already shown promising results [78]. Recent human clinical trials focused their attention mainly on Leber's Hereditary Optic Neuropathy (LHON); nevertheless, many animal studies about other optic neuropathies and RGCs neuroprotection have been conducted. Animal studies showed promising results about regeneration and neuroprotection. Concerning LHON, it has been shown that intravitreal injections of AAV2-ND4 (adeno-associated viruses type 2 carrying NADH dehydrogenase, subunit 4 gene) viral vector are safe and feasible [79]. However, long-term efficacy and risks such as tumors are concerns still to be better considered.

The use of neurotrophic factors is promising, but limited, as there are not yet adequate techniques to selectively target these molecules at specific sites. Moreover, they may be beneficial for some types of cells, but toxic for others if they reach certain concentration values; they also have a reduced half-life. The solution to these challenges seems to be the design of carrier particles that preserve the neuroprotective molecules and direct them to the desired targets.

### 3.7. Rehabilitation Therapy

Rehabilitation treatment is a therapeutic method that aims to reeducate the patient to the use of residual vision through repeated visual stimulation. What this technique is based on is the neuronal plasticity of the visual system, as the damaged nerve fibers are able to reorganize themselves and repair the injury by creating new connections or rediscovering existing but little exploited networks. Therefore, if the optic nerve is completely damaged (e.g., very late stages of glaucoma), it is impossible to apply this strategy. An example of neuronal plasticity concerning the visual system is the phenomenon of blindsight, which occurs in some patients suffering from cortical blindness: Weiskrants et al. [80] described it as the capacity of some patients to respond to visual stimuli in the corresponding area of the visual field without perceiving it consciously. This phenomenon may be attributed to the recruitment of subcortical pathways in order to partially compensate for the loss of visual functionality.

Many methods have been studied and tested to improve vision in partially blind patients. These methods include vision training exercises such as computer-based vision restoration therapy [2], retinal implants, and noninvasive brain current stimulation. In the latter case, direct current or alternating current stimulation (ACS) can be used to improve brain excitability or resynchronize neuronal oscillations. ACS uses weak current pulses delivered through electrodes placed on the forehead for some minutes daily, for a period of 10 days on average. Electroencephalography and functional magnetic resonance demonstrated local activation of the visual cortex, reorganization of the neural pathways, and enhanced blood flow in the stimulated area. ACS showed proregenerative effects in controlled trials in patients with glaucoma and optic neuropathy.

The process of physiological plasticity can be enhanced by neurorehabilitation cycles, which can be further supported by the utilization of neurotrophic factors.

Other technologies indicated in this field are epiretinal electrode implants, which stimulate RGCs, subretinal electrodes, transchoroidal implants, devices acting on the optic nerve, and cortical implants, which target the brain areas responsible for vision. The latter could be the most suitable therapeutic strategy to target the latest stages of glaucoma, where no function of the optic nerve is left [81, 82]. However, although the results are promising, the ultimate goal of restoring good vision is still almost a mirage, and several limitations and problems still have to be overcome in order to give these new technologies a clinical application. Regarding epiretinal implants, all the studies described left the patients far below the limit of legal blindness (20/200) and all the stimuli were unable to maintain specific retinotopy. Moreover, this is an invasive approach, highly anatomically destructive, and without the possibility of recovery in case of device failure. Subretinal implants, when compared to the previous ones, are more stable as they are implanted beneath the retina, they do not require connection to external devices, and their stimulation thresholds are lower. Nevertheless, this technology is still invasive and provides a minimal or no visual recovery. Transchoroidal implants are less invasive but require a higher stimulation. The possibility of acting directly on the optic nerve should theoretically allow stimulating both the central and peripheral visual field with a lower intensity of the stimuli and lower invasiveness since the electrodes are localized in a smaller area. Several kinds of implants have been proposed [83], which permitted the perception of light and spatial orientation in a small number of cases. However, it has been shown that with time the intensity of the stimuli required becomes higher, and this is probably due to the development of gliosis surrounding the implant after time. Finally, cortical implants showed encouraging results in the study of Dobelle [84], but in order to decrease the intensity of stimulation thresholds, penetrative cortical implants should be used, which would lead to higher invasiveness, risk of infection, and inflammation with reactive gliosis and neuronal death.

Thus, subsequent studies are needed to fully understand the mechanisms that are implied and to refine these devices.

## 4. Conclusions

Glaucoma is an increasingly widespread social disease and many advances have been achieved to improve the diagnostic and therapeutic resources available (Table 1). However, new options need to be further enhanced and supported by significant experimental data on the biological responses of intraocular and brain tissues, in particular trabecular cells, RGCs, retinal fibers, and optical pathways. The union between clinic, biology, and biotechnology and their synchronous enforcement appears to be the winning strategy to defeat the “silent thief of sight”: the challenge is still open.

## Figures and Tables

**Table 1:** Summary of the new treatment strategies under development.

	Anatomic target	Mechanism of action	Expected effect	Advantages	Disadvantages
Electrical stimulation	Trabecular meshwork (TM)	(i) Relaxation of TM, with less resistance to aqueous humor outflow	(i) Significant effect on lowering IOP	(i) Early stages of glaucoma (ii) Facilitates the physiological pathway of the aqueous humor through TM towards Schlemm's canal (SC)	(i) Not in advanced stages of glaucoma (ii) Needs to be repeated

Micropulse cyclophotocoagulation (CPC) and ultrasound cyclomodification	Ciliary body	(i) Decrease the secretory activity of the ciliary epithelium	(i) Same IOP lowering effect as traditional CPC diode	(i) Advanced stages of glaucoma (ii) Minimal heat diffusion (iii) Less pain than traditional CPC	(i) Not in the early stages of glaucoma

Mesenchymal stem cells (MSCs)	Retinal ganglion cells (RGCs)	(i) Differentiation into retinal cell types (RGC) (ii) production of growth factors	(i) Neuroprotective effects	(i) Pluripotency (ii) Ease of extraction (bone marrow, adipose tissue) (iii) Autologous transplantation	(i) Ethical issues

Exosomes	RGCs	(i) Translation of new proteins through mi-RNA-dependent mechanisms (ii) RGC survival and preservation of function	(i) Neuroprotective effects	(i) Easily isolated and purified (ii) Do not proliferate (iii) Easily stored (iv) Able to migrate (v) Immunologically inert	(i) Not clear which dose for a therapeutic effect (weekly, biweekly, or monthly)

Optic nerve scaffolds	Optic nerve	(i) Stimulation and regeneration of damaged nerve fibers	(i) Neural regeneration	(i) Potential restoration of neural function	(i) Obstacles to neural regeneration: apoptosis of RGC, difficulty in triggering the axonal growth, inhibitory factors

ROCK inhibitors	Optic nerve and retinal ganglion cells	(i) Positive regulation of neural growth triggers	(i) Neural growth	(i) Augmented RGC survival	(i) Few studies to support this evidence

Neurotrophic factors (NF)	Optic nerve and retinal ganglion cells	(i) Interaction with macrophage-derived factors with immunomodulation	(i) Neural regeneration and growth	(i) Autologous molecules	(i) Local inflammation is needed to induce secretion of NF (ii) Not selective (iii) Reduced half-life

Alternating current stimulation (ACS)	Visual cortex and neural vision pathways	(i) Weak current pulses delivered to the brain	(i) Improvement of brain excitability and resynchronization of neuronal oscillation	(i) Very advanced glaucoma stages	(i) Few studies to support this evidence

Epiretinal, subretinal, and transchoroidal electrode implants	Optic nerve and RGCs	(i) Weak current pulses delivered to the eye	(i) Improvement of the optic nerve and retinal ganglion cell excitability	(i) Very advanced glaucoma stages (ii) Low stimulation thresholds	(i) Invasive approach (ii) Gliosis over the implant over time (iii) Poor results described in the literature

## Data Availability

All necessary data are included within the article.
